# Assessing the effectiveness of problem-based learning in physical diagnostics education in China: a meta-analysis

**DOI:** 10.1038/srep36279

**Published:** 2016-11-03

**Authors:** Jianmiao Wang, Yongjian Xu, Xiansheng Liu, Weining Xiong, Jungang Xie, Jianping Zhao

**Affiliations:** 1 Teaching and Research Section of Diagnostics, Tongji Hospital, Tongji Medical College, Huazhong University of Science and Technology, 1095 Jiefang Road, Wuhan 430030, China; 2 Department of Respiratory and Critical Care Medicine, Tongji Hospital, Tongji Medical College, Huazhong University of Science and Technology, 1095 Jiefang Road, Wuhan 430030, China

## Abstract

Problem-based learning (PBL) has been extensively applied as an experimental educational method in Chinese medical schools over the past decade. A meta-analysis was performed to assess the effectiveness of PBL on students’ learning outcomes in physical diagnostics education. Related databases were searched for eligible studies evaluating the effects of PBL compared to traditional teaching on students’ knowledge and/or skill scores of physical diagnostics. Standardized mean difference (SMD) with 95% confidence interval (CI) was estimated. Thirteen studies with a total of 2086 medical students were included in this meta-analysis. All of these studies provided usable data on knowledge scores, and the pooled analysis showed a significant difference in favor of PBL compared to the traditional teaching (SMD = 0.76, 95%CI = 0.33–1.19). Ten studies provided usable data on skill scores, and a significant difference in favor of PBL was also observed (SMD = 1.46, 95%CI = 0.89–2.02). Statistically similar results were obtained in the sensitivity analysis, and there was no significant evidence of publication bias. These results suggested that PBL in physical diagnostics education in China appeared to be more effective than traditional teaching method in improving knowledge and skills.

Problem-based learning (PBL) is an educational approach usually characterized by the use of real problems as a context for students to acquire knowledge and develop skills^1^. This pedagogy was developed from educational innovation to resolve dissatisfaction with the conventional medical education practices in the late 1960s^2^. It has since been implemented in many medical school curricula and other professional training programs around the world.

Unlike traditional teaching model, PBL approach is student-centered. Students learn about a subject through the experience of problem solving in a tutor-led small group. Many studies have suggested that students in a PBL curriculum become better at problem solving and self-directed learning than those in a traditional curriculum^3^,^4^,^5^. However, there is also evidence indicating that PBL teaching model is not superior to the traditional approach with respect to the acquisition of factual knowledge^1^,^6^,^7^, and the effects of PBL may be different in different subjects.

As a bridge course in medical education, physical diagnostics is very important for medical students to acquire basic clinical knowledge and develop clinical skills. The latter are usually defined as clinical reasoning and clinical problem solving that are mostly related to taking an adequate medical history, conducting an appropriate physical examination and establishing a proper diagnosis^8^. Although the physical diagnostics course structure has been changed to the PBL approach in many medical schools^9^, it is still not clear whether PBL in physical diagnostics education is superior to traditional pedagogies such as lecture-based learning and system-based approach.

In China, PBL application in medical education is not a routine pedagogy for many reasons^10^. The development of PBL is behind that in other countries. In recent years, due to current shifts in approaches to medical education prevalent in China, this teaching model has been extensively applied as an experimental educational method in many medical curricula including physical diagnostics^11^,^12^. However, the results of PBL studies about physical diagnostics education in China have been inconclusive or inconsistent. Considering the relatively small sample size of most studies, it is possible to perform a quantitative synthesis of the evidence with rigorous methods. The aim of current meta-analysis was to evaluate the overall effectiveness of PBL compared to traditional teaching in Chinese physical diagnostics education.

## Results

### Search results

**Figure 1:**
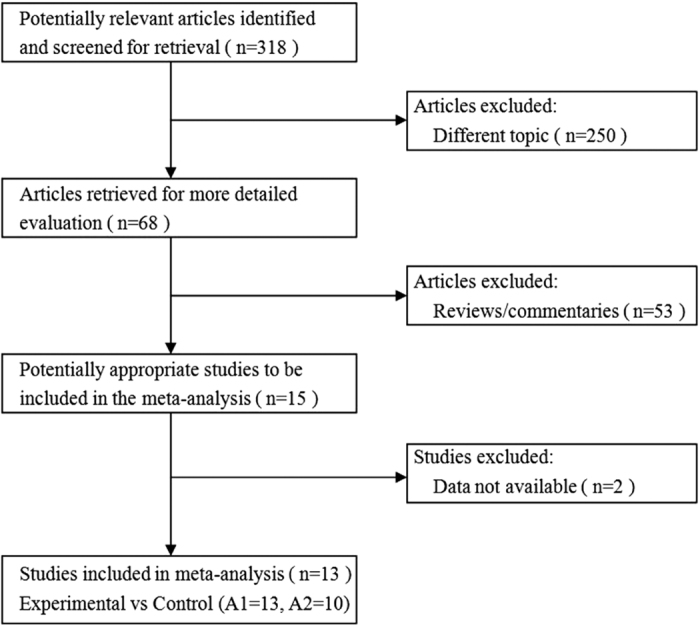
Flowchart for identification of studies. A: The number of studies that provided usable data on knowledge scores (A1) and skill scores (A2).

The flowchart for identification of studies is shown in Fig. 1. At the stage of identification, we searched the related databases using the search strategy described in the Methods section. In total, 318 potentially relevant articles were identified. At the screening stage, we excluded 250 articles with different topics, and also eliminated 53 reviews and commentaries. Fifteen studies were potentially appropriate and assessed for eligibility according to the criteria described in the Methods section, of which two studies were excluded because they did not provide sufficient data^12^,^13^. Thus, a total of 13 controlled studies, involving 2086 medical students, met the inclusion criteria and were selected for this meta-analysis^14^,^15^,^16^,^17^,^18^,^19^,^20^,^21^,^22^,^23^,^24^,^25^,^26^.

### Study characteristics

The characteristics of these 13 included studies are shown in Table 1. All of them were published in Chinese between 2007 and 2014, and evaluated the effects of PBL compared to traditional teaching in physical diagnostics courses (also named clinical diagnostics in China). The sample sizes ranged from 20 to 230 students in PBL group and 25 to 229 in control group. The major of students in almost all studies was clinical medicine. The school system was different, however nearly half of the studies were carried out in the five-year system medical students. Nine studies described that the students in each group were matched for the basic medical knowledge test scores or college entrance exam scores. The duration of study was one semester, which was mentioned in 9 studies. Both knowledge scores and skill scores of physical diagnostics were used as outcome measures in all studies. The scores in the knowledge exam of physical diagnostics were used to assess how well the students mastered the related theoretical knowledge, and the scores in the practical skill test of physical diagnostics were used to evaluate the students’ clinical skills including history taking, physical examination and diagnostic reasoning.

### Study quality

**Figure 2:**
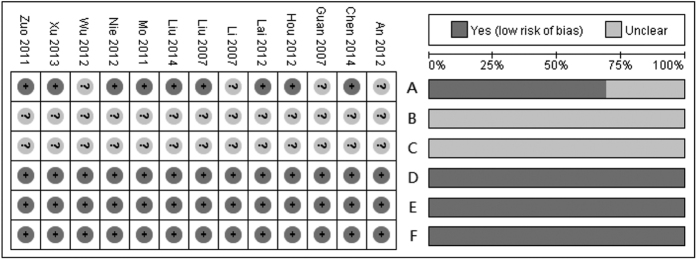
Summary of each methodological quality item for each included study and graph of each quality item presented as percentages across all included studies. A: Randomized? B: Allocation concealment? C: Blinding? D: Incomplete outcome data addressed? E: Free of selective reporting? F: Free of other bias?

The summary of the methodological quality of each study is shown in the left panel of Fig. 2, and the graph of each quality item presented as percentages across all studies is shown in the right panel. Nine studies were designed as randomized controlled trials, and the other 4 studies were unclear. All studies reported complete outcome data and were free of selective reporting and other bias. The allocation concealment and blinding were not stated in these studies.

### Effects of PBL on knowledge scores

**Figure 3:**
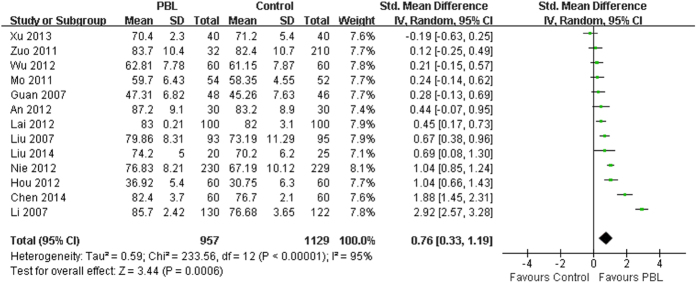
Forest plot for the effects of PBL on knowledge scores compared with the traditional teaching.

The effects of PBL compared to the traditional teaching on knowledge scores were reported in all 13 included studies involving 2086 medical students (PBL group = 957, control group = 1129). Seven studies suggested that there was a statistically significant difference between the PBL and the control group in students’ knowledge scores. Because a high degree of heterogeneity was observed across all of the 13 studies (I^2^ = 95%, P < 0.001), the random-effects model was applied for the meta-analysis. The analytical results showed a significant difference in knowledge scores (SMD = 0.76, 95%CI [0.33, 1.19], P = 0.0006) in favor of PBL, compared with the traditional teaching (Fig. 3). The summary effects also favored the PBL group when using the fixed-effects model (SMD = 0.81, 95%CI [0.72, 0.91], P < 0.001). In addition, we performed a sensitivity analysis by excluding individual studies sequentially. Statistically similar results were obtained (SMD = 0.58~0.84, P < 0.01), suggesting the stability of this meta-analysis. In the stratified analysis by school system, we pooled the data from 6 studies in which target population were all five-year system students using the random-effects model (I^2^ = 97%, P < 0.001). A statistically significant difference was also observed in knowledge scores (SMD = 0.90, 95%CI [0.04, 1.76], P = 0.04) in favor of PBL, compared with the traditional teaching.

### Effects of PBL on skill scores

**Figure 4:**
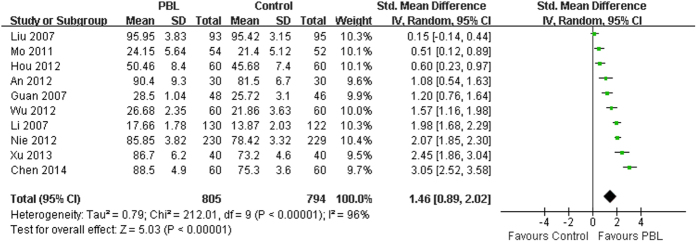
Forest plot for the effects of PBL on skill scores compared with the traditional teaching.

The effects of PBL compared to the traditional teaching on skill scores were reported in all included studies. Ten studies involving 1599 medical students (PBL group = 805, control group = 794) provided usable data for this analysis. Because a high degree of heterogeneity was observed across all of the 10 studies (I^2^ = 96%, P < 0.001), the random-effects model was applied for the meta-analysis. The analytical results showed a significant difference in skill scores (SMD = 1.46, 95%CI [0.89, 2.02], P < 0.001) in favor of PBL, compared with the traditional teaching (Fig. 4). The summary effects also favored the PBL group when using the fixed-effects model (SMD = 1.40, 95%CI [1.29, 1.51], P < 0.001). In addition, we performed a sensitivity analysis by excluding individual studies sequentially. Statistically similar results were obtained (SMD = 1.28~1.60, P < 0.01), suggesting the stability of this meta-analysis. In the stratified analysis by school system, we pooled the data from 5 studies in which target population were all five-year system students using the random-effects model (I^2^ = 92%, P < 0.001). A statistically significant difference was also observed in skill scores (SMD = 1.08, 95%CI [0.47, 1.69], P = 0.0006) in favor of PBL, compared with the traditional teaching.

### Publication bias

**Figure 5:**
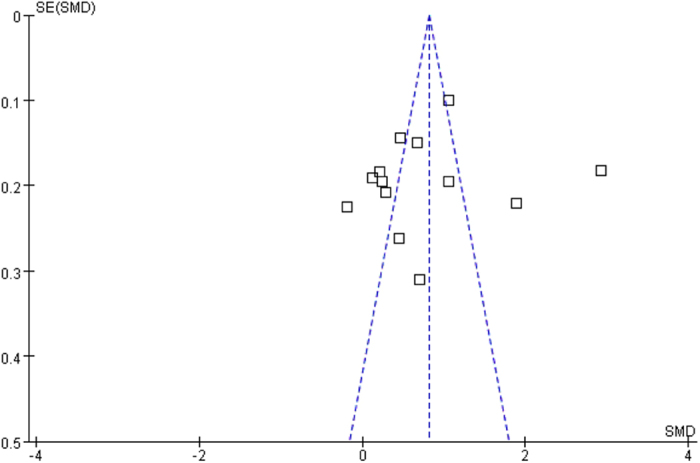
Funnel plots for the assessment of potential publication bias in knowledge scores.

**Figure 6:**
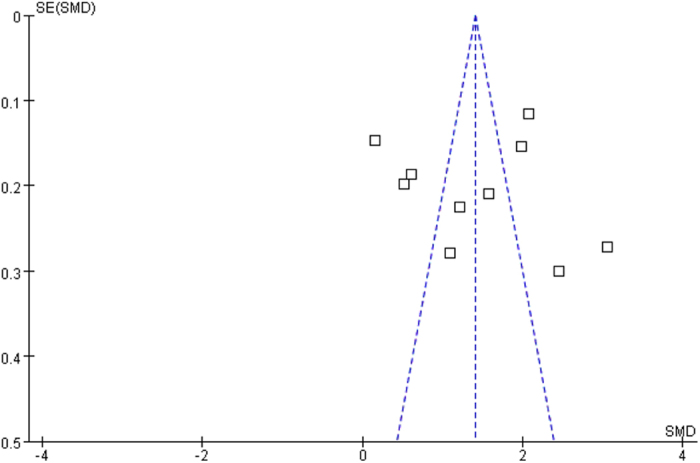
Funnel plots for the assessment of potential publication bias in skill scores.

Funnel plots for knowledge scores (Fig. 5) showed no clear evidence of publication bias, and the test using Egger’s method did not suggest publication bias, either (t = −0.47, P = 0.649). Funnel plots for skill scores (Fig. 6) also showed no clear evidence of publication bias, and the test using Egger’s method did not suggest publication bias, either (t = 0.14, P = 0.896).

## Discussion

Although PBL has been widely adopted in medical education all over the world, the application of this teaching method in China is still at the elementary stage. Given that different education systems or cultural backgrounds could influence the effectiveness of PBL method^27^, the target population should be limited to Chinese medical students in the Chinese education system in order to evaluate the potential effectiveness of the PBL approach in China. This meta-analysis of studies in the field of Chinese physical diagnostics education assessed whether there were differences in quantitative outcomes between PBL pedagogy and the traditional teaching method.

The results of current meta-analysis show that the students in the PBL groups have better knowledge scores than those in the traditional teaching method groups, which are consistent with the findings of recent PBL studies in Chinese pediatric, pharmaceutical and dental education^10^,^28^,^29^. However, in western countries, some education researchers have questioned the performance of PBL students in the knowledge exams and have argued that PBL has a negative effect on the acquisition of factual knowledge^1^,^6^,^7^. Given the differences in higher medical education between China and the western countries, there are several possible reasons for the inconsistent results. Firstly, PBL pedagogy is totally different from the traditional teaching method in which students achieve knowledge passively from their teachers. Chinese students have received the traditional teaching model since primary education. PBL teaching model is a novelty for them and has greatly stimulated their interest in learning^30^. Secondly, most Chinese medical schools use unified textbooks and syllabuses for all students, which is very different from the western countries. In the present study, both PBL and traditional students used the same textbook and syllabus of physical diagnostics. PBL students appeared to be more proactive in learning, which led them to get higher knowledge scores. Thirdly, teachers and other faculty personnel in a PBL program usually engage more with students^31^. Chinese teachers always emphasize the importance of course examinations and the keen pursuit of high scores promotes the students to excel in the exams^30^.

Despite the explosion of technological advances, the basic clinical skills of history taking, physical examination and counseling remain the most important and effective diagnostic tools^8^. Therefore, the development of above skills in the physical diagnostics education is considered crucial for medical students. According to the results of our study, there is a robust positive effect from PBL on the skill scores of students, and no single study reports negative effects. These results are consistent with the previous findings that PBL can effectively enhance clinical skills^1^,^32^,^33^,^34^,^35^. The use of problems as a vehicle for developing problem solving skills is one of the key characteristics of PBL, while the important aspects of physical diagnostics education involve the development of students’ clinical diagnostic reasoning skills and ability to solve clinical problems, which represent the advantages of PBL. Hence, the superiority of PBL relative to the traditional teaching method could be more evident when considering the clinical skills.

Obviously, there is a high heterogeneity among the included studies. In the stratified analysis by school system, significant heterogeneity also exists, which might have resulted from the following factors. The definition and implementation of PBL could vary widely among medical schools and educators^36^. The educational quality of different medical schools in China could be very uneven, and the medical students from different schools could have different learning background. There are still no unified criteria for the evaluation of the effectiveness of PBL on knowledge and skills. In addition, many other factors that are difficult to measure and control could also affect the success of PBL^37^. Therefore, the random-effects model was applied in this meta-analysis. The high levels of heterogeneity in the effect sizes indicate that more than one population is generating the effect sizes and that the present mean effect sizes are not broadly generalizable. Further research that focuses on moderator variables is required to reduce and resolve the heterogeneity.

It is generally accepted that randomized controlled trials provide the highest level of evidence for the effectiveness of an intervention. However, it is not always possible to use a very rigorous randomized controlled trial approach for educational research. Although most of the studies included in this meta-analysis were designed as randomized controlled trials, none of them described the allocation concealment or blinding method. Overall, the methodological quality of the included studies is not high. In the analysis of knowledge scores (Fig. 3), two of the effect sizes are very high and the mean effect size is large. Similarly, five of the effect sizes are high in the analysis of skill scores (Fig. 4), and the mean effect size is also quite large. Although statistically significant results were also obtained after removing those corresponding studies (data not shown), moderating variable analyses were not performed due to limited studies. Thus, the results of current meta-analysis need to be interpreted with caution. In addition, some of the included studies also used questionnaire surveys as an additional measurement to assess the effectiveness of PBL in physical diagnostics education. However surveys are limited in reliability and validity, subjective and prone to rater biases, and we failed to perform the meta-analysis of those outcomes due to inadequate data.

In summary, the present meta-analysis shows that PBL in physical diagnostics education in China appears to be more effective than traditional teaching method in improving knowledge and skills. Based on the limitations of this meta-analysis, we believe that further well-designed studies on this topic are needed.

## Methods

### Data sources

We searched China National Knowledge Infrastructure (CNKI), China Science Periodical Database (CSPD), Chinese BioMedical Literature Database (CBM) and English computerized databases including PubMed, EMBASE and Cochrane Database using the following terms: (PBL OR (problem-based learning)) AND (diagnostics OR diagnosis). There were no language restrictions. Searches were current as of April 2016. References of all primary studies and review articles were reviewed for additional references.

### Study selection

Included studies met the following criteria: (1) target population: medical students in Chinese medical schools; (2) study design: controlled trials in physical diagnostics education; (3) interventions: PBL teaching in the experimental group and traditional teaching in the control group; (4) outcome measurements: students’ knowledge scores and/or skill scores of physical diagnostics. In addition, we excluded studies with insufficient data for calculating effect sizes.

### Data extraction and quality assessment

Two reviewers independently identified trials and reviewed the titles, abstracts and citations (Jianmiao Wang and Jianping Zhao). Two reviewers independently assessed studies for inclusion based on the criteria for population, intervention, study design and outcome measurements (Jungang Xie and Xiansheng Liu). Data were also independently extracted by two reviewers (Yongjian Xu and Weining Xiong). From each study, the following information was extracted: first author’s surname, publication year, course name, sample size, student characteristics, intervention method, duration of study and outcomes. We also contacted study authors for missing data. The methodological quality of each study was evaluated with the risk of bias table according to the Cochrane Collaboration.

### Statistical analysis

Standardized mean difference (SMD) for continuous outcomes, with 95% confidence interval (CI), was calculated for each study. Studies were then pooled together using SMD as appropriate. The Z-test was used for overall effect and two-sided P < 0.05 was considered as statistically significant. The Q-statistic was calculated to examine result heterogeneity among studies, and P < 0.10 was considered significant. The fixed-effects model was used when the effects were assumed to be homogenous; otherwise, the random-effects model was applied. The I^2^ statistic was also calculated to efficiently test for the heterogeneity, with I^2^ < 25%, 25–75% and >75% to represent low, moderate and high degree of inconsistency, respectively^38^. Publication bias was examined in funnel plots and tested with Egger’s weighted regression method^39^. The meta-analysis was performed using Review Manager 5.0.23 (Cochrane Library Software, Oxford, UK) and STATA 12.0 (STATA Corporation, College Station, Texas, USA).

## Additional Information

**How to cite this article**: Wang, J. *et al*. Assessing the effectiveness of problem-based learning in physical diagnostics education in China: a meta-analysis. *Sci. Rep.* **6**, 36279; doi: 10.1038/srep36279 (2016).

**Publisher’s note:** Springer Nature remains neutral with regard to jurisdictional claims in published maps and institutional affiliations.

## Figures and Tables

**Table 1:** Characteristics of published studies included in this meta-analysis.

Study	Course name	Number of students (E/C)	Major of students	School system	Students matched for	Educational approach (E/C)	Duration of study	Outcome measures
An 2012	Clinical diagnostics	30/30	Clinical medicine	Five-year system	Age, Sex, ES	PBL/Traditional teaching	One semester	KS, SS
Chen 2014	Physical diagnostics	60/60	Medicine	Not described	Age, Sex, BS	PBL/Traditional teaching	Unclear	KS, SS
Guan 2007	Clinical diagnostics	48/46	Clinical medicine	Five-year system	Not described	PBL/Traditional teaching	One semester	KS, SS
Hou 2012	Physical diagnostics	60/60	Clinical medicine	Five-year system	BS	PBL/Traditional teaching	Unclear	KS, SS
Lai 2012	Physical diagnostics	100/100	Clinical medicine	Five-year system	Not described	PBL/Traditional teaching	One semester	KS, SS
Li 2007	Physical diagnostics	130/122	Clinical medicine	Five-year system	BS	PBL/Traditional teaching	One semester	KS, SS
Liu 2007	Physical diagnostics	93/95	Clinical medicine	Three-year system	Age, Sex, ES	PBL/Traditional teaching	One semester	KS, SS
Liu 2014	Physical diagnostics	20/25	Clinical medicine	Mixed	Not described	PBL/Traditional teaching	Unclear	KS, SS
Mo 2011	Physical diagnostics	54/52	Clinical medicine	Five-year system	Not described	PBL/Traditional teaching	One semester	KS, SS
Nie 2012	Physical diagnostics	230/229	Clinical medicine	Three-year system	Age, Sex, ES	PBL/Traditional teaching	One semester	KS, SS
Wu 2012	Clinical diagnostics	60/60	Clinical medicine	Mixed	BS	PBL/Traditional teaching	One semester	KS, SS
Xu 2013	Physical diagnostics	40/40	Clinical medicine	Not described	Age, Sex, ES	PBL/Traditional teaching	Unclear	KS, SS
Zuo 2011	Clinical diagnostics	32/210	Clinical medicine	Eight-year system	Sex, BS	PBL/Traditional teaching	One semester	KS, SS

E/C, experimental group/control group; ES, college entrance exam scores; PBL, problem-based learning; KS, knowledge scores; SS, skill scores; BS, basic medical knowledge test scores.

## References

[b1] Albanese M. A. & Mitchell S. Problem-based learning: a review of literature on its outcomes and implementation issues. Acad. Med. 68, 52–81 (1993). 8447896 10.1097/00001888-199301000-00012

[b2] Neufeld V. R. & Barrows H. S. The “McMaster Philosophy”: an approach to medical education. J. Med. Educ. 49, 1040–1050 (1974). 4444006

[b3] Jones R., Higgs R., de Angelis C. & Prideaux D. Changing face of medical curricula. Lancet. 357, 699–703 (2001). 11247568 10.1016/S0140-6736(00)04134-9

[b4] Kermaniyan F., Mehdizadeh M., Iravani S., Markazi Moghadam N. & Shayan S. Comparing lecture and problem-based learning methods in teaching limb anatomy to first year medical students. Iran. J. Med. Educ. 7, 379–388 (2008).

[b5] Enarson C. & Cariaga-Lo L. Influence of curriculum type on student performance in the United States Medical Licensing Examination Step 1 and Step 2 exams: problem-based learning vs. lecture-based curriculum. Med. Educ. 35, 1050–1055 (2001). 11703641 10.1046/j.1365-2923.2001.01058.x

[b6] Dochy F., Segers M., Van den Bossche P. & Gijbels D. Effects of problem-based learning: a meta-analysis. Learn. Instr. 13, 533–568 (2003).

[b7] Vernon D. T. & Blake R. L. Does problem-based learning work? A meta-analysis of evaluative research. Acad. Med. 68, 550–563 (1993). 8323649 10.1097/00001888-199307000-00015

[b8] Holmboe E. S. Faculty and the observation of trainees’ clinical skills: problems and opportunities. Acad. Med. 79, 16–22 (2004). 14690992 10.1097/00001888-200401000-00006

[b9] Donkers K., Garrubba C., Daniel L. & Ennulat C. Perceptions of physician assistant students’ readiness with system-based vs. problem-based physical diagnosis curriculum. Internet J. Allied Health Sci. Pract. 13, 1–8 (2015).

[b10] Huang B., Zheng L., Li C., Li L. & Yu H. Effectiveness of problem-based learning in Chinese dental education: a meta-analysis. J. Dent. Educ. 77, 377–383 (2013). 23486905

[b11] Zhang Y. . The effectiveness of the problem-based learning teaching model for use in introductory Chinese undergraduate medical courses: a systematic review and meta-analysis. PLoS One. 10, e0120884 (2015). 25822653 10.1371/journal.pone.0120884 PMC4378971

[b12] Rui Z. . Preliminary investigation into application of problem-based learning in the practical teaching of diagnostics. Adv. Med. Educ. Pract. 6, 223–229 (2015). 25848334 10.2147/AMEP.S78893 PMC4378870

[b13] Shen H. L., Lin H. S. & Wang S. W. A tentative study of the application of problem-based learning teaching model in the diagnostics education [in Chinese]. Dig. Manage. Sci. 18, 32–33 (2009).

[b14] An L. H., Wang C. Y., Yao Y. H. & Zhao H. Application of problem-based learning in teaching of diagnostics [in Chinese]. Chin. Higher Med. Educ. 10, 105–106 (2012).

[b15] Chen J. J. & He Y. S. Application of problem-based learning teaching model in the diagnostics education [in Chinese]. J. Front. Med. 34, 35–36 (2014).

[b16] Guan B., Fan M. J., Zhao G. F. & He Q. Application of problem-discussing teaching method in clinical diagnostics [in Chinese]. J. Kunming Med. Coll. 28, 234–236 (2007).

[b17] Hou W. P., Yuan F. H., Wang Y. Q., Guo Y. H. & Li Y. PBL applied in internship teaching of diagnostic signs [in Chinese]. Northwest Med. Educ. 20, 594–596 (2012).

[b18] Lai Y., Zeng J. & Chen D. B. Design and practice of PBL teaching in physical diagnosis [in Chinese]. Chin. Higher Med. Educ. 10, 16–17 (2012).

[b19] Li X. D., Xu R. X., Wei L. P. & Li W. F. Initiative teaching method applied in teaching diagnostics [in Chinese]. Mod. Hosp. 7, 121–123 (2007).

[b20] Liu F. P., Huang L., Zhou W. F., Gao X. & Liu X. The application of problem-based learning in the teaching of diagnostics [in Chinese]. Chin. J. Med. Educ. 27, 78–79 (2007).

[b21] Liu A. Q. . Application of problem-based learning teaching model in the physical diagnostics education [in Chinese]. Basic Med. Educ. 16, 382–385 (2014).

[b22] Mo X. N. & Xin N. Application and cogitation of problem-based learning teaching model in diagnostics education [in Chinese]. Res. Integr. Tradit. Chin. West. Med. 3, 45–47 (2011).

[b23] Nie J. R., Wen S. Q., Yang L. J., Jin J. & Yan Y. P. Exploration of PBL teaching model application in diagnostics education [in Chinese]. Health Vocat. Educ. 30, 69–70 (2012).

[b24] Wu H. N. . Application of problem-based learning method in clinical diagnostics teaching [in Chinese]. Chin. J. Med. Educ. 32, 547–549 (2012).

[b25] Xu G. Y., Zhang J. Y. & Li Z. F. Application and discussion of PBL teaching method in the diagnostics education during the novitiate of respiratory system [in Chinese]. Guide Chin. Med. 11, 746–748 (2013).

[b26] Zuo C. . Preliminary exploration of the application of problem-based learning in diagnostic practice teaching [in Chinese]. Chin. J. Evidence-Based Med. 11, 1098–1102 (2011).

[b27] Frambach J. M., Driessen E. W., Chan L. C. & van der Vleuten C. P. Rethinking the globalisation of problem-based learning: how culture challenges self-directed learning. Med. Educ. 46, 738–747 (2012). 22803751 10.1111/j.1365-2923.2012.04290.x

[b28] Gao X. . Effects of problem-based learning in paediatric education in China: A meta-analysis. J. Evid. Based Med. doi: 10.1111/jebm.12190 (2016). 26845692

[b29] Zhou J. . Effectiveness of problem-based learning in Chinese pharmacy education: a meta-analysis. BMC Med. Educ. 16, 23 (2016). 26787019 10.1186/s12909-016-0546-z PMC4719679

[b30] Ren X., Yin J., Wang B. & Roy Schwarz M. A descriptive analysis of medical education in China. Med. Teach. 30, 667–672 (2008). 18777425 10.1080/01421590802155100

[b31] Hamdy H. & Agamy E. Is running a problem-based learning curriculum more expensive than a traditional Subject-Based Curriculum? Med. Teach. 33, e509–e514 (2011). 21854146 10.3109/0142159X.2011.599451

[b32] Neville A. J. Problem-based learning and medical education forty years on. A review of its effects on knowledge and clinical performance. Med. Princ. Pract. 18, 1–9 (2009). 19060483 10.1159/000163038

[b33] Scaffa M. E. & Wooster D. M. Effects of problem-based learning on clinical reasoning in occupational therapy. Am. J. Occup. Ther. 58, 333–336 (2004). 15202631 10.5014/ajot.58.3.333

[b34] Hoffman K., Hosokawa M., Blake R. Jr., Headrick L. & Johnson G. Problem-based learning outcomes: ten years of experience at the University of Missouri-Columbia School of Medicine. Acad. Med. 81, 617–625 (2006). 16799282 10.1097/01.ACM.0000232411.97399.c6

[b35] Koh G. C., Khoo H. E., Wong M. L. & Koh D. The effects of problem-based learning during medical school on physician competency: a systematic review. CMAJ. 178, 34–41 (2008). 18166729 10.1503/cmaj.070565 PMC2151117

[b36] Maudsley G. Do we all mean the same thing by “problem-based learning”? A review of the concepts and a formulation of the ground rules. Acad. Med. 74, 178–185 (1999). 10065058 10.1097/00001888-199902000-00016

[b37] Wood D. F. ABC of learning and teaching in medicine: problem based learning. BMJ. 326, 328–330 (2003). 12574050 10.1136/bmj.326.7384.328 PMC1125189

[b38] Higgins J. P., Thompson S. G., Deeks J. J. & Altman D. G. Measuring inconsistency in meta-analyses. BMJ. 327, 557–560 (2003). 12958120 10.1136/bmj.327.7414.557 PMC192859

[b39] Egger M., Davey Smith G., Schneider M. & Minder C. Bias in meta-analysis detected by a simple, graphical test. BMJ. 315, 629–634 (1997). 9310563 10.1136/bmj.315.7109.629 PMC2127453

